# The Roles of Regulatory B Cells in Cancer

**DOI:** 10.1155/2014/215471

**Published:** 2014-06-02

**Authors:** Yan He, Hongyan Qian, Yuan Liu, Lihua Duan, Yan Li, Guixiu Shi

**Affiliations:** Department of Rheumatology and Clinical Immunology, The First Affiliated Hospital of Xiamen University, Xiamen 361003, China

## Abstract

Regulatory B cells (Bregs), a newly described subset of B cells, have been proved to play a suppressive role in immune system. Bregs can inhibit other immune cells through cytokines secretion and antigen presentation, which give them the role in the pathogenesis of autoimmune diseases and cancers. There are no clear criteria to identify Bregs; different markers were used in the different experimental conditions. Massive researches had described the functions of immune cells such as regulatory T cells (Tregs), dendritic cells (DCs), and B cells in the autoimmune disorder diseases and cancers. More and more researches focused on the roles of Bregs and the cytokines such as Interleukin-10 (IL-10) and transforming growth factor beta (TGF-**β**) secreted by Bregs. The aim of this review is to summarize the characteristics of Bregs and the roles of Bregs in cancer.

## 1. Introduction 

The relationship between immune system and cancer development has been well described over the past several decades. The immune system plays an important role in the prevention of tumors. It could specially identify and eliminate tumor cells through the tumor-associated antigens (TAA) or molecules expressed by the tumor cells [1]. This process was described as tumor immune surveillance or tumor immunoediting, which was divided into three essential phases: elimination, equilibrium, and escape. In the first phase, elimination, the transformed cells were recognized and eliminated by the innate immune response such as nature killer cells (NK) and macrophages before they became clinically apparent. The second phase was an equilibrium phase between the tumor cells and immunity. The antigen specific T cells could induce the adaptive immune response. The tumor cells would decrease the tumor-specific antigens and even lose the major MHC-I and MHC-II antigens along with the tumor progression. Finally, in the escape phase, the tumor cells became less immunogenic, escaped from immune attack, and suppressed antitumor immune response leading to the tumor production and growth [2]. Cancer genesis was the result of the immune escape. The immune effector cells and the cytokines played a key role in pursuing each phase. They had been clarified by the roles played in cancer immune surveillance [3]. In recent years, literatures have found some new subsets of the immune cells which are relevant to the tumor immune surveillance and promote the cancer production and progression, for example, the Tregs and Bregs. In this review, we focus on the relationship between the regulatory B cells and their functions in cancer.

## 2. Characteristics of Regulatory B Cells

### 2.1. Phenotypes and Markers of Bregs

In recent years, B cells have been demonstrated to downregulate inflammatory reactions and induce tolerance by production of IL-10 and/or TGF-*β* and interacting with pathogenic T cells to inhibit harmful immune responses.

The term “regulatory B cells” was introduced by Mizoguchi and collaborators, who identified Bregs as an IL-10-producing B cell subset in 2002 [4]. Those Bregs had been shown to ameliorate murine allergic and autoimmune diseases, such as contact hypersensitivity (CHS) [5], asthma [6], experimental autoimmune encephalomyelitis (EAE) [7], lupus [8], and collagen induced arthritis (CIA) [9]. Topical studies in CIA had identified the transitional 2 marginal-zone precursor (T2-MZP) cells that played an immunosuppressive function both* in vivo* and* in vitro* [10, 11]. To date, there are no precise unique phenotype markers to identify Bregs. Markers on mouse Bregs resembled those on CD1d^hi^CD5^+^ B10 cells [5], CD1d^hi^ MLN B cells (B220^+^CD1d^hi^CD21^int⁡^CD62^low^IgM^int⁡^CD23^int⁡^) [12], CD1d^hi^CD21^hi^CD23^−^CD24^hi^IgM^hi^IgD^lo^ marginal-zone B cells, CD19^+^CD21^hi^CD23^hi^CD24^hi^IgD^hi^IgM^hi^CD1d^hi^ T2-MZP cells [13], and Tim-1^+^ Bregs [14]. IL-15 coupled to granulocyte macrophage colony stimulating factor could convent naïve splenic B cells into IL-10-producing B cells. Those Bregs shared common markers with B10 cells and T2-MZP Bregs and acquired the expression of CD138 but lost the expression of CD19 [15]. Differing from above regulatory B cell subsets, the surface characteristics of adipose Bregs were CD1d^lo^CD5^−/lo^CD11b^lo^CD21/CD35^lo^CD23^−/lo  ^CD25^+^CD69^+^CD72^hi^CD185^−^CD196^+^IgM^+^IgD^+^ [16]. These Bregs could maintain adipose tissue homeostasis and limit obesity-associated inflammation. The IL-10-producing B cell subset characterized in humans normally represents 1% to 3% of spleen B cells and <1% of peripheral blood B cells [17]. Human regulatory B cells were enriched in both transitional (CD24^hi^CD38^hi^) [18] and memory (CD24^hi^CD27^+^) [17] B cells. IL-10 production by CD24^hi^CD27^+^ B cells regulated monocyte tumor necrosis factor alpha (TNF-*α*) production [17]. CD19^+^CD24^hi^CD38^hi^ B cells inhibited proinflammatory cytokine production by CD4^+^ T cells, dependent on IL-10, CD80, and CD86 but not TGF-*β* [18]. Human CD19^+^CD25^hi^CD86^hi^CD1d^hi^ B regulatory cells could suppress the proliferation of CD4^+^T cells and enhance Foxp3 and cytotoxic T-lymphocyte antigen 4 (CTLA-4) expression in Treg cells by producing IL-10 and TGF-*β* [19]. Bregs did not belong to any clearly defined B cell subsets but they added value in both the CD27^+^ and the CD38^hi^ compartments [20]. Regardless of the different markers used to identify Bregs, the majority of protective effects of Bregs are dependent on IL-10 [4, 5, 7, 18, 21], a potent deactivator, which limits the intensity and duration of inflammatory responses. Thus, IL-10 secretion is still a vital standard in the identification of Bregs.

A few of signal pathways were under the responsibility of the production of IL-10 by Bregs. It had been demonstrated that the Breg response could be promoted by stimulation with Toll-like receptor 4 (TLR4) and Toll-like receptor 9 (TLR9) ligands [22]. LPS with PIM (PMA+ionomycin+monensin) could induce B10 cells* in vitro* [5]. Using a mouse model for multiple sclerosis, B10 cells maturation into functional IL-10-secreting effector cells that inhibited autoimmune diseases* in vivo* required IL-21 and CD40-dependent cognate interactions with T cells [23]. IL-21 induced GrB^+^ human Bregs expressing high levels of GrB, which thereby limited T cell proliferation by a GrB-dependent degradation of the T cell receptor *ζ*-chain [24]. Agonistic anti-CD40 specifically targeted T2 B cells and enriched Bregs upon short-term* in vitro* culture [11]. MyD88 was thought to be involved but not critical to the development of Breg, while played a considerate role in IL-10 expression [20]. B cell linker protein, as a signaling component for Bregs function, was essential for the suppression of CHS and EAE by mediating IL-10 production [25]. Nuclear factor *κ*B-*α*-kinase (NF*κ*B) and signal transducer and activator of transcription 3 (STAT3) were involved in the secretion of IL-10 by Bregs [26]. Matsumoto and colleagues had found that the production of IL-10 was reduced in Bregs from mice with stromal interaction molecule 1 (STIM1) and stromal interaction molecule 2 (STIM2) depleted [27].

### 2.2. The Roles of Bregs in Immune System

Regulatory B cells performed a diversity of mechanisms to regulate immune responses and target many different immune cell types, such as DCs [28] and macrophages [29] as well as T helper 1 (Th1) cells and T helper 2 (Th2) cells [30]. It has been demonstrated that Bregs were capable of suppressing the proliferation of CD4^+^  CD25^−^  T cells [31] and production of interferon gamma (IFN-*γ*) and IL-17 by Th1 and T helper 17 (Th17) cells, respectively [32, 33].* In vitro* studies in human had further postulated the potential ability of Breg cells to influence innate immunity by abrogating mitogen-stimulated secretion of TNF-*α* by monocytes, macrophages, and T cells. Yet regulatory B cells had no impact on the secretion of IL-6 and IL-8 by CD4^+^  T cells [34]. Mean B10 and progenitor B10 cell frequencies from patients with autoimmune disease were significantly higher than controls after CD40L with LPS/CpG stimulation [17]. These suppressive effects were mediated by IL-10. A number of studies indicated that the production of IL-10 by Bregs in mice and human was important for generation of at least two regulatory T- cell subtypes and conventional Treg cells as well as type 1 regulatory T cells (Tr1) [32–34]. The lack of Bregs resulted in a decrease of Foxp3^+^ Tregs [33]. B cell deficiency caused a significant reduction in the number of peripheral but not thymic Tregs. Adoptive transfer of WT B cells into *μ*MT mice restored both Treg numbers and recovery from EAE [35]. B cells isolated from donor MRL/lpr mice and stimulated with agonistic anti-CD40* in vitro* converted autologous effector T cells into Tr1 cells [11]. Coculture of CD4^+^ T cells by IL-10^+^ B cells announced that IL-10^+^ B cells were in a position to induce CD4^+^T cells to produce large quantity of IL-10, which mediated the immunosuppression and protection from development of cerebral malaria [31]. IL-10 produced by Bregs was essential for the generation and maintenance of the pool of Tregs. Bregs induced pulmonary infiltration of CD4^+^CD25^+^Foxp3^+^ conventional Tregs, which controlled allergic airway inflammation [13]. As a result, activated Bregs could directly or indirectly target immune cells to regulate immune responses.

## 3. Regulatory B Cells in Cancer

### 3.1. B Cells in Cancer

Both positive and negative roles of B cells during tumor immunity have been reported. Depletion of CD20-expressing B cells increased tumor burden in the lungs of mice intravenously injected with B16-F10 melanoma [36]. B cells facilitated T-mediated responses, which in turn impaired tumor development [36, 37]. The induction of CD4^+^ and CD8^+^ T cells was significantly impaired in B cell-depleted mice with B16 melanoma tumors [37]. Activated B cells could mediate significant tumor regression in an IgG2b-dependent manner [36, 38]. These studies highlighted the effector function of B cells as a source of IgG2b which were highly cytotoxic toward tumor cells [38].

Despite these, negative regulatory functions of B cells during immune responses to tumors have also been proposed. As we know, the mutation and accumulation of B cells and the antibodies secreted by B cells may play decisive roles in the tumor formation. Studies on B cell-deficient *μ*MT mice showed that B cell deficiency enhanced CD4^+^ T cell priming and helped for CD8^+^ T cell-mediated tumor immunity [39]. The repertoire of CD4^+^ helper T cells was limited in the presence of B cells, resulting in reduced antitumor immune responses [40]. Upon stimulation by irradiated tumor cells, the production of IFN-*γ* from CD8^+^ T cells and NK cells was found to be markedly increased in the B cell-deficient as compared with wild-type conditions [41]. Murine EMT-6 mammary tumors grew readily in immune competent mice but poorly in B-cell-deficient mice [42]. Syngeneic tumors progressed poorly in *μ*MT mice deficient in B cells unless replenished with B220^+^ B cells [40, 43]. B cells could exert lots of functions to promote cancer, such as producing immunoglobulins and cytokines that could induce Fc receptors (FcR) and complement mediated chronic inflammation, whereas the chronic inflammation was required for carcinogenesis [44–46]. B cells produced TGF-*β*, thereby mediating suppression of cellular immune responses. Previously, studies showed that B cells required IL-10 and TNF-*α* to promote tumorigenic. IL-10 and TNF-*α* probably could mediate Th2 activation and inhibition of cytotoxic activity of CD8^+^  T cells and nucleus. The cytokines such as TNF-*α* produced by B cells could activate the inhibitor of *κ*B-*α*-kinase (I*κ*B*α*), signal transducer, and activator of STAT3 in tumor cells, which would get a relapse of the castration resistant prostate cancer [47]. Moreover, the studies of the tumor growth in a mouse model of skin carcinogenesis had shown that the immune cells infiltrating in the premalignant lesions would reduce in B cell deficiency mice, which resulted in the inhibition of skin carcinoma development. Antibodies secreted by B cells could form the immune complexes (ICs) that would stimulate myeloid cells recruitment at tumor sites, which promoted the tumor growth via binding to the Fc*γ*-activating receptors. Also, the macrophages would become protumor myeloid cells [44, 48]. A similar role of B cells was observed in a synthetic mammary cancer model. It was reported that the antitumor immune response by T cells was enhanced in mice with B cell deficiency in this cancer model [40]. It also supported that the tumor was reduced by treatment with anti-mouse IgM/IgG antibodies, which would deplete the mutation B cells [49].

### 3.2. Bregs in Cancer

Bregs, as a separate subpopulation of B cells, have been mainly researched in their roles in the autoimmune diseases and inflammatory diseases [50]. Considering the tumor promoting role of the B cells and the immune regulating role of the Bregs, recent studies have focused on the role of Bregs in cancer. Previous studies had illustrated that the tumor inhibition effect of T cells was regulated by IL-10 production, which threw out a point that Bregs might be involved in cancer [41].

Also, IL-10 was a persistent regulatory cytokine which could repress Th1 and Th2 cytokine expression [51]. A recent study showed that Bregs could still secrete antibodies especially with IgM subtype as the activated plasmablast/plasma B cells. Immunoglobulins could also mediate inflammation to promote cancer as described above [44, 52].

Prevacid B and coworkers had described a unique subset of Breg cells in the studies with the mouse 4T1 model of breast cancer. The Bregs belonged to the CD19^+^ B220^+^ CD25^+^ B2 lymphocytes which were needed in the progress of 4T1 murine breast cancer cells metastasis lung [53]. Since the CD25 is highly expressed on all activated T cells, B cells, and the thymic Tregs, the authors described this novel subpopulation of Bregs as tBregs. This research had found that the proportion of tBregs was significantly increased in peripheral blood and secondary lymphoid organs. 4T1 murine cancer cells could directly induce the generation of tBregs that inhibited the proliferation of nonactive and preactivated T cells. Similar role of tBregs was proved in the human cancers* in vitro* [54]. tBregs highly expressing TGF-*β* as well as CD40, CD86, and MHC I and II molecules could promote the generation of Foxp3^+^ Tregs. The conversion of CD4^+^  T cells into Tregs was relying on cell contact between T and B cells and the TGF-*β* secretion. In the process of lung metastasis of mammary adenocarcinoma 4T1 cancer cells, Tregs were needed in order to inhibit antitumor defenses of NK cells [55]. In conclusion, the researcher had proved that the tBregs could transform nonregulatory CD4^+^ T cells (non-Tregs) to active Tregs through secreting the TGF-*β*, which in turn inhibited T cells proliferation and increased tumor metastasis [53, 54]. Also, cancer cells could convert normal B cells into tBregs. So, as long as cancer persists, the cancer cells would induce tBregs generation and inhibit the antitumor immune process [43, 56]. Recently, it was reported that nonmetastatic cancer cells expressed and utilized metabolites of the 5-lipoxygenase (5-LO) pathway to induce tBregs generation [57]. The presence of inhibitors of 5-LO/FLAP (5-LO activating protein) significantly reduced the expression of almost every tBreg-associated marker, such as decreased expression of CD25, CD81, BAFFR, and B7-H1 and phosphorylation of STAT3. Functionally, these cells also failed to suppress proliferation of T cells and did not induce conversion of FoxP3^+^CD4^+^ Tregs from non-Treg CD4^+^ T cells [57]. This was the first clearly defined example reported on the existence and function of Bregs in cancer.

The anti-CD20 antibody reduction that could deplete B cells was proved to be effective in the treatment of non-Hodgkin lymphomas and CLL, but some patients showed resistance to anti-CD20 therapy or eventually relapse. A recent study has revealed that the presence of Bregs and their IL-10 might inhibit the therapy efficacy [58]. B cell depletion by CD20 antibody would greatly enhance cancer progression and metastasis. Both murine and human tBregs expressed low levels of CD20 and, as such, anti-CD20 mostly enriched for these cells [59]. In the study of lymphoma, the model mice received anti-CD20 treatment and then, following transfer of Bregs, resulted in tumor burden significantly increased [58, 60]. Bregs could affect the phagocytic capacity of macrophages, both* in vivo* and* in vitro* [58]. Myeloid cells and macrophages were responsible for clearance of anti-CD20 bound to tumor cells [60].

In the study of 7,12-dimethylbenzanthracene (DMBA) and 12-O-tetradecanoylphorbol-13-acetate (TPA) induced skin carcinogenesis, the cancer growth was reduced in B cell deficiency mice (Rag2^−/−^ mice) [61]. However, it would be partially rescued with transfer of B cells. Nonetheless, transfer of B cells could not promote the skin cancers in Rag2^−/−^ mice when the mice were deficient of TNF-*α*. TNF-*α* knockout mice transferred of B cells could not enhance the skin cancer either. The number of CD19^+^CD21^+^IL-10-secreting B cells which were defined as Bregs showed significant decrease in TNF-*α* KO mice. The results above suggested that Bregs could promote cancer growth and the secretion of TNF-*α* might lead to the generation and accumulation of Bregs in cancer.

Recently, study found that IL-21 induced GrB-expressing Breg cells resided within the microenvironment of different tumor types including breast, ovarian, cervical, colorectal, and prostate carcinomas [24]. GrB^+^ B cells might contribute to the modulation of cellular adaptive immune responses by Treg-like mechanisms, possibly allowing the escape of certain tumors from an efficient antitumor immune response [24].

## 4. Conclusion

Both cancer escape and autoimmune diseases belonged to the inappropriate regulatory immune process. B cells mediated humoral immunity by secreting antibody and also regulated T cell maturation and function via serving as APCs and providing regulatory molecules [62]. The functions of B cells in autoimmunity and cancer diseases were well described. Lots of evidences had suggested the promoting tumor role of B cells, probably through inhibiting the activation of T cells, especially the CD8^+^ T cells. In recent years, a distinct subpopulation of B cells that performed significant immunosuppressive has been described. Despite the fact that more and more literatures focused on the regulatory B cells, their clear phenotype and characteristic markers are still not definite. Since all the Bregs could secrete IL-10 and the majority of protective effects of Bregs required IL-10, IL-10 secreting B cells was considered as Bregs. The phenotype and naming of human and mouse Bregs in different experimental conditions were displayed in Table 1.

Bregs, as a subset of B cells, could exert B cells function such as secreting antibody and cytokines, which induced the IC production and stimulated cell signal resulting in tumor progression. Bregs as well as total B cells could promote cancer growth mainly by inhibiting the cytotoxic activity of Th1/CD8+ cells. This process was actually mediated by their IL-10 and TGF-*β* production.

As we discussed above in all mouse cancer models, Bregs, together with other components of immune system, promoted tumor progression. IL-10 produced by Bregs could reduce the Th1/CD8^+^ cells. Furthermore, TGF-*β* secreted by Bregs could convert CD4^+^  T cells into Tregs that would promote tumor progression. In studies of anticancer therapies by anti-CD20 therapy, Bregs and their IL-10 should be responsible for the development of lymphoma resistance to anti-CD20 therapy.

There are still some subjects of Bregs that remain to be established. Whether Bregs can affect other immune cells and whether different subset of Bregs can differentially participate in immune modulation have not been illustrated. Also, further studies are needed to research on tumor treatment through targeting Bregs.

## Figures and Tables

**Table 1:** The immunophenotype and naming of the regulatory B cells in mouse and human.

Species	Marker/phenotype	Naming	References
Human	CD24^hi^ CD27^+^	B10 cells	[17]
	CD19^+^ CD25^hi^ CD86^hi^ CD1d^hi^	Bregs	[19]
	CD19^+^ CD24^hi^ CD38^hi^	Bregs	[18]
	CD19^+^ CD38^+^ CD1d^+^ IgM^+^ CD147^+^ GrB^+^	GrB^+^ Bregs	[24]

Mouse	CD19^+^ CD5^+^ CD1d^hi^	B10 cells	[5]
	B220^+^ CD21^int^ CD62^int^ IgM^int^ CD23^hi^ CD1d^hi^	CD1d^hi^ MLN B cells	[12]
	CD1d^hi^ CD21^hi^ CD23^−^ CD24^hi^ IgM^hi^ IgD^lo^	Marginal zone B cells	[13]
	CD19^+^ CD21^hi^ CD23^hi^ CD24^hi^ IgD^hi^ IgM^hi^ CD1d^hi^	Transitional 2 marginal-zone precursor (T2-MZP)	[13]
	Tim-1^+^	Tim-1^+^ Bregs	[14]
	CD1d^lo^CD5^−/lo^ CD11b^lo^ CD2 1/CD35^lo^CD23^−/lo^ CD25^+^ CD69^+^ CD72^hi^ CD185^−^ CD196^+^ IgM^+^ IgD^+^	Adipose IL-10^+^ B cells	[16]
	CD19^+^ B220^+^ CD25^+^	Tumor-evoked regulatory B cells (tBregs)	[53]
